# The Drugs of New York

**Published:** 1888-04

**Authors:** 


### *THE DRUGS OF NEW YORK.*

The *New York World* has been recently conducting a series of analyses, from which it deduces the conclusion that nearly forty per cent. of the drugs sold in New York City were below the standard required by the Pharmacopœia.

"In a few cases, drugs were found to be of an inferior quality, notably ipecac., in which the medicinal portion of the drug had entirely disappeared; senna, which contained decayed leaves possessing no value; gentian, long kept and worm-eaten; taraxacum, aconite and rhubarb, inferior specimens when gathered.

"The most important adulteration discovered was in the tinctures of nux vomica and opium—articles over which the retail druggist has absolute control. In the matter of crude drugs, he is often at the mercy of the wholesaler, but in pharmaceutical preparations like the above, he, and he only, is accountable for whatever deficiency may exist. He can purchase powdered opium and powdered nux vomica of unquestionable value, and, in the case of the former at least, samples which have previously been assayed and have their alkaloidal strength printed on the label. From these, by following the directions of the Pharmacopœia, he can readily produce tinctures which shall absolutely correspond to the officinal requirements."

